# Michigan State Veterinary Medical Association

**Published:** 1897-04

**Authors:** William Jopling

**Affiliations:** Secretary


Secretary.

### MICHIGAN STATE VETERINARY MEDICAL ASSOCIATION.

THE fifteenth annual meeting was called to order at 2.30 P.M., Tuesday, February 2, 1897, in the parlors of the New Grand Hotel, Lansing, Michigan, President Dr. W. W. Thorburn in the chair. The following were present: Drs. J. Hawkins, D. G. Sutherland, D. Cummings, J. A. Dell, E. A. A. Grange, J. W. Ferguson, W. J. Byers, J. C. Whitney, Wm. Jopling, W. W. Thorburn, J. J. Joy, W. A. Giffen, James Drury, W. M. Burdick, H. M. Gohn, Wallace McQueen, Judson Black, G. W. Dunphy, A. McKercher, B. C. McBeth, S. Brenton, and George E. Metcalf. Drs. Wm. S. Hamilton, of Chelsea; D. M. Waldo, of Grand Ledge; L. A. Grimell, of North Lansing; and E. T. Handy, of Eaton Rapids, were present as visitors and became members of the Association.

The President, Dr. Thorburn, gave a ringing address. Among other things, he said that the year just closed had been one of deep concern to the veterinary profession. Statistics show that since 1892 the valuation of horses in Michigan has made a 50 per cent. decline, and he felt assured that each member present had felt the effects of it in their practice. He gave encouragement by saying that he thought the darkest days are past and we are soon to emerge into the light and sunshine of professional prosperity, and the trials through which we have recently passed will enhance the sweetness and pleasure of the future. He felt only pity and regret for those who, in the trials of financial depression, have taken false steps by entering upon quackery and questionable methods of practice. He suggested the advisability of a table of rates for professional services. On veterinary legislation he spoke at some length, and thought the only way to secure the passage of a bill was to insert a "time" clause. A vote of thanks was tendered President Thorburn for his very able address, which was ordered spread on the minutes.

The minutes of last meeting were read and approved. The report of the Secretary for the year ending February 2, 1897, was read and approved. The Treasurer's report for the year ending February 2, 1897, was read and referred to the Finance Committee.

The Committee on Intelligence and Education made a verbal report, which gave rise to quite a discussion, particularly that calling attention to the vivisection bill in the District of Columbia. The committee was instructed to draft resolutions disapproving such a bill becoming a law. The report of the committee was accepted.

The Committee on Diseases made a verbal report, which was accepted.

The report of the Committee on Legislation, owing to the death of the chairman, Dr. Rutherford, was made by Dr. Giffen, of Detroit. The principal point at issue was the presenting to the Legislature a bill with time clause inserted. A yea and nay vote of the members disclosed the fact that the majority of the Association did not favor a time clause. The report was accepted.

A committee was appointed by the President, consisting of Drs. E. A. A. Grange, G. W. Dunphy, and J. A. Dell, to draft resolutions on the death of Dr. J. D. Rutherford.

Meeting adjourned until 7.30 P.M. Called to order for evening session at 8 o'clock.

Committee on Intelligence and Education presented resolutions in reference to the vivisection bill, which, upon motion, were approved and the Secretary instructed to send printed copies to the Senators and Congressmen of this State.

The Finance Committee reported that they found everything correct with the Treasurer's books; report accepted.

Upon motion, the President appointed a committee, consisting of Drs. J. Hawkins, J. W. Ferguson, and W. McQueen, to communicate with his honor, Governor Pingree, requesting him to retain Prof. E A. A. Grange as State Veterinarian.

Moved and supported that this Association donate $5.00 to the Pasteur Monument Fund, providing we have funds enough in the treasury after paying expenses. Motion lost.

Prof. Grange gave a very able, interesting, and instructive talk on the "Milk Apparatus and its Diseases," which was illustrated by charts.

Moved and supported and carried that Prof. Grange be allowed to invite any of the students from the Agricultural College who may wish to see surgical operations to be present at the same.

Dr. J. Black read a paper prepared by Dr. Stevens, of Yale, giving twenty experiments with barium chloride in the treatment of obstinate constipation and acute indigestion. Dr. Stevens speaks very highly of the remedy.

Meeting adjourned at 11.30 P.M., to meet at Dr. Thorburn's infirmary at 9 A.M., to witness surgical operations.

Wednesday, February 3d, 9 A.M.: As prearranged, all the members of the Association present and several students from the Agricultural College met at Dr. Thorburn's to witness operations. Dr. S. Brenton, of Detroit, was the chief operator, and performed median neurectomy for injured tendons and disease of the fetlock, tarsal tenotomy for spavin, and neurotomy for navicular disease and ringbone. The operations proved very interesting, and Dr. Brenton was tendered a hearty vote of thanks for the valuable instructions given.

Meeting called to order again at 2 P.M. The election of officers was taken up and resulted as follows: President, Dr. A. Campbell, Jackson; First Vice-President, Dr. J. Black, Richmond; Second Vice-President, Dr. W. A. Giffen, Detroit; Third Vice-President, Dr. H. M. Gohn, St. Johns; Secretary and Treasurer, Dr. William Jopling, Owosso; Directors, Drs. J. J. Jay, Chairman, Detroit; Dr. W. M. Burdock, Chesaning; Dr. A. McKercher, Lansing; Dr. J. Byers, Charlotte; Dr. J. W. Ferguson, Bay City; Dr. James Drury, Ypsilanti.

Moved and supported that a bill be drafted and presented to the Legislature now in session to protect the "College Title of Veterinary Surgeon;" carried.

Drs. G. W. Dunphy, H. M. Gohn, and S. Brenton promised papers for the next annual meeting.

A vote of thanks was given Dr. Thorburn, the retiring President, for the interest taken in the Association and for his courtesy to the members while in Lansing, his home.

A vote of thanks was extended our host and hostess for their uniform kindness and attention.

The following standing committees were appointed:

Intelligence and Education: Drs. E. A. A. Grange, Lansing; S. Brenton, Detroit; G. W. Dunphy, Quincy.

Committee on Diseases: Drs. D. Cummings, Port Huron; J. Hawkins, Detroit; H. M. Gohn, St. Johns.

Committee on Finance: Drs. W. W. Thorburn, Lansing; Wallace McQueen, Oxford; George E. Metcalf, Detroit.

Committee on Legislation: Drs. W. A. Giffen, Detroit; J. W. Ferguson, Bay City; Judson Black, Richmond.

Adjourned in due form. WILLIAM JOPLING, Secretary.

